# Remdesivir-Associated Acute Liver Failure in a COVID-19 Patient: A Case Report and Literature Review

**DOI:** 10.7759/cureus.34221

**Published:** 2023-01-26

**Authors:** Mohammad A Ahmed-Khan, George Matar, Kyle Coombes, Kayvon Moin, Bishoy M Joseph, Carly M Funk

**Affiliations:** 1 Faculty of Medicine, Danbury Hospital - Yale School of Medicine, Danbury, USA; 2 School of Medicine, American University of the Caribbean, Cupecoy, SXM

**Keywords:** drug-induced liver injury (dili), acute liver failure (alf), hepatology, pulmonology, remdesivir, covid-19

## Abstract

There is a broad classification of the causes of acute liver failure (ALF) that include drug-induced liver injury (DILI). In this report, we aim to discuss the association between remdesivir, a novel therapeutic drug for hypoxic coronavirus disease 2019 (COVID-19) pneumonia, and DILI with subsequent ALF in a patient who was recently treated with the drug in question. Remdesivir, which is a direct-acting nucleoside RNA polymerase inhibitor, is one of the only FDA-approved drugs on the market for COVID-19 pneumonia associated with hypoxia. Our case describes a patient with an extensive past medical history who was treated for COVID-19 pneumonia with remdesivir and subsequently developed ALF in the absence of all other possible etiologies. This association has only been highlighted in anecdotal case reports in the past and to a lesser degree in the safety documentation of remdesivir.

## Introduction

Acute liver failure (ALF) is a life-threatening illness that mainly occurs in patients with no pre-existing liver disease [1]. It is characterized by an acute course (less than 26 weeks), acute liver injury (markedly elevated transaminases), impaired synthetic function (international normalized ratio (INR) ≥1.5), and clinical signs of hepatic encephalopathy (altered mental status, asterixis, etc) [2]. Etiologies for this condition include viral hepatitis, autoimmune hepatitis, steatohepatitis, ischemic hepatopathy, and drug-induced liver injury (DILI) [3]. In the United States, DILI is the most common etiology of ALF, with acetaminophen as the most common causative agent [4]; however, many other drugs may cause DILI and lead to ALF, as listed by the National Institute of Diabetes and Digestive and Kidney Diseases (NIDDK) [5]. Our case describes a patient who was diagnosed with COVID-19 pneumonia and was prescribed remdesivir, and shortly thereafter, developed ALF in the absence of all other possible etiologies. 

## Case presentation

A 69-year-old female with a past medical history of atrial fibrillation (A-fib) treated with apixaban, insulin-dependent diabetes mellitus (IDDM), stage 4 chronic kidney disease (CKD), heart failure with preserved ejection fraction (HFpEF), obstructive sleep apnea (OSA), chronic obstructive pulmonary disease (COPD), pulmonary hypertension, hypertension (HTN), hyperlipidemia (HLD), bipolar disorder, and depression was seen in the emergency department (ED) for upper respiratory symptoms, peripheral cyanosis, productive cough, headaches, and myalgias. She was found to be positive for severe acute respiratory syndrome coronavirus 2 (SARS-CoV-2) RNA by polymerase chain reaction (PCR) testing and was admitted to the medical service for further management with supportive therapy and remdesivir (200 mg loading dose on presentation followed by maintenance dosing 100 mg daily until the day of discharge). On hospital day four, her symptoms had resolved, remdesivir was discontinued, and she was discharged home with a tapering dose of prednisone. The patient had a down-trending white blood cell count from the time of admission, her kidney function was at baseline, and the remainder of her labs were within normal limits. 

She subsequently returned to the ED two days later with worsening weakness, shortness of breath (SOB), and hypoxia. Upon reevaluation, she was found to have new-onset transaminitis, encephalopathy, and an INR > 1.5 (Table 1). She was readmitted with the working diagnosis of ALF in the setting of DILI with an R-factor of 4.2, indicating a mixed hepatocellular-cholestatic pattern of liver injury. Workups for alternative causes, including infectious causes, ethanol, acetaminophen, hepatitis, and ischemia were all negative. The infectious workup included Ebstein-Barr virus and cytomegalovirus, which were both negative for acute infection; blood cultures were also obtained and were negative. A toxicology panel was ordered, which resulted in undetectable ethanol levels and an acetaminophen level of <5. The hepatitis workup included hepatitis A total Ab, hepatitis B core (HBc) total Ab, hepatitis B surface antigen (HBsAg) screen, and hepatitis C virus (HCV)antibodies, which were all nonreactive. Ischemic hepatitis was ruled out as the patient had remained hemodynamically stable with no signs of shock during her initial hospitalization and at the time of readmission. An abdominal doppler was also performed to rule out ischemia and Budd-Chiari syndrome, which showed normal hepatopetal portal venous flow without liver congestion. This information further cemented the diagnosis of DILI with subsequent ALF in the setting of remdesivir exposure for COVID-19 pneumonia treatment. 

**Table 1:** Laboratory trends during second admission ALT: alanine aminotransaminase; AST: aspartate aminotransaminase; Alk Phos: alkaline phosphatase; T. Bili: total bilirubin; INR: international normalized ratio

Time since Admission (hours)	ALT (U/L)	AST (U/L)	Alk Phos (U/L)	T. Bili (mg/dL)	INR
0	237	268	171	1.2	-
12	298	338	156	1.5	2.98
20	377	421	152	1.9	3.09
25	443	532	164	2.3	3.73
35	1,347	2,050	154	2.4	12.4

**Figure 1:**
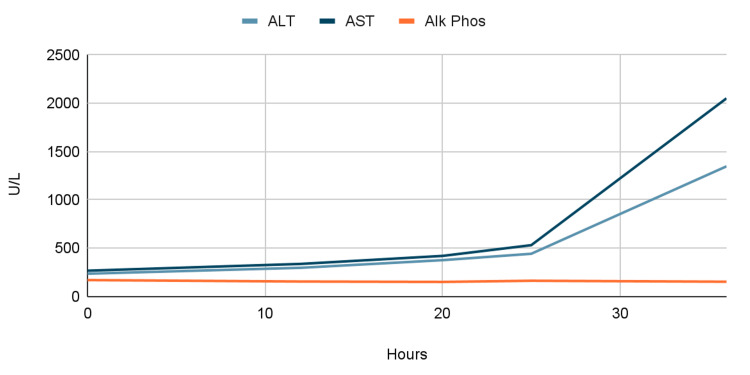
Graph of laboratory trends during second admission: liver function tests by time since admission ALT: alanine aminotransaminase; AST: aspartate aminotransaminase; Alk Phos: alkaline phosphatase

**Figure 2:**
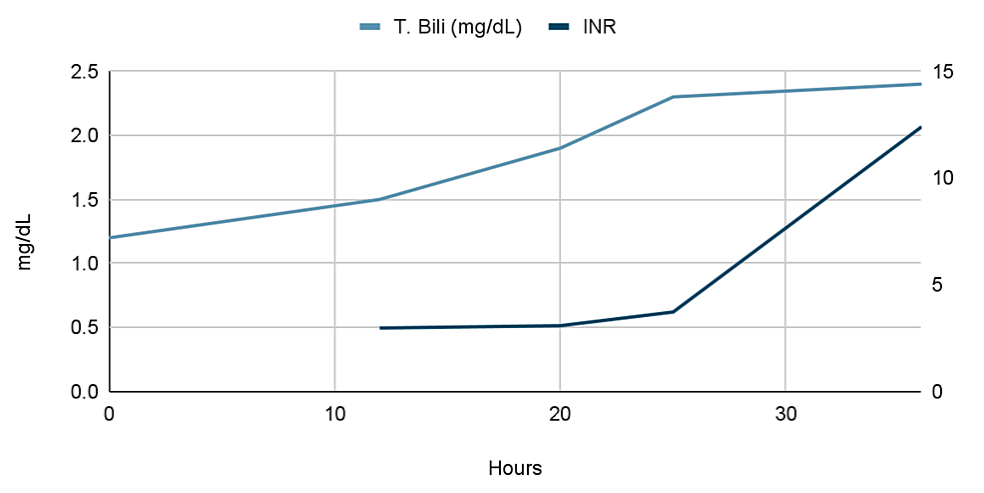
Graph of laboratory trends during second admission: liver function tests by time since admission T. Bili: total bilirubin; INR: international normalized ratio

Given this information and the concern for rising liver function tests (LFTs) (Table 1, Figure 1, Figure 2), the patient was started on intravenous (IV) N-acetylcysteine (NAC). This was done similarly to the acetaminophen toxicity protocol, which is administered in three separate doses. An initial loading dose of 150 mg/kg was given over 15-60 minutes followed by a dose of 50 mg/kg over four hours, and finally, a dose of 100 mg/kg was given over 16 hours for a total of 300 mg/kg given over 20-21 hours. At this point, the case was discussed with the liver center at a nearby hospital and the decision was made to transfer the patient to the intensive care unit (ICU) at said hospital. However, the patient became more encephalopathic and later unresponsive without a palpable pulse, requiring advanced cardiovascular life support and cardiopulmonary resuscitation with IV epinephrine. The patient was successfully resuscitated and subsequently intubated and transferred to the ICU. The patient later succumbed to her illness after being transferred to a nearby hospital.

## Discussion

This patient, whose COVID-19 symptoms initially resolved with remdesivir treatment, was readmitted only two days after initial discharge with new onset transaminitis consistent with a mixed hepatocellular-cholestatic pattern of liver injury. The main patterns of liver injury include hepatocellular, cholestatic, and mixed hepatocellular-cholestatic and all three can be seen in DILI. In the hepatocellular pattern of liver injury, the serum aspartate aminotransaminase (AST) and alanine aminotransaminase (ALT) are elevated out of proportion to the elevation in alkaline phosphatase or gamma-glutamyl transpeptidase (GGT). The cholestatic pattern of liver injury presents with markedly elevated alkaline phosphatase and GGT out of proportion to the elevations in ALT and AST. The mixed hepatocellular-cholestatic pattern of liver injury, which is the pattern most commonly seen in DILI, is characterized by more proportional elevations in serum alkaline phosphatase and ALT [6].

DILI can be defined as liver injury caused by medications that lead to liver dysfunction and abnormal liver tests with the reasonable exclusion of other etiologies [7]. There are three main forms of DILI, which are direct, idiosyncratic, and indirect hepatotoxicity. The direct hepatotoxicity form of DILI is caused by medications that are intrinsically toxic to the liver in a dose-dependent manner. Medications in the idiosyncratic hepatotoxicity category have little to no intrinsic toxicity to the liver; however, they are still implicated in liver injury in rare cases often due to metabolites. Lastly, medications with indirect hepatotoxicity may cause DILI due to the action of the drug instead of intrinsic toxicity [8]. The exact mechanism by which remdesivir causes liver injury is unknown; however, it may be in the setting of direct hepatocyte toxicity due to mitochondrial RNA polymerase inhibition. Remdesivir may also be subject to drug-drug interactions in the liver as it is a substrate for the CYP 3A4 enzyme, as well as, the OATP1B1 and p-glycoprotein transporters [9]. 

Pharmacokinetic data has demonstrated that remdesivir and remdesivir triphosphate, its active precursor, are primarily renally eliminated (74%) in individuals with normal kidney function [10]. There is conflicting data regarding the use and safety of remdesivir in individuals with CKD and acute kidney injury (AKI). Jamale et al. conducted a study where 46 patients with either CKD or AKI were treated with remdesivir for COVID-19 infections. They reported that the treatment was well-tolerated as there were no clinically significant increases in ALT as well as no side effects requiring early discontinuation of treatment seen in all 46 individuals [11]. Cheng et al. performed a study on critically ill patients with COVID-19 and an estimated glomerular filtration rate (eGFR) less than 30 mL/min1.73 m^2^ where 34 individuals received remdesivir treatment, and 25 received standard care. They reported that there was no significant difference in kidney toxicity, transaminitis, need for invasive mechanical ventilation, new dialysis, or mortality between groups [12]. Other studies, however, have reported that remdesivir use in patients with impaired renal function may have a negative risk-benefit ratio and discourage routine use in this patient population [13]. There is accumulating evidence of both the safety and efficacy of remdesivir use in patients with impaired kidney function although more large controlled studies are necessary. 

NAC is well known to be a mainstay of treatment for acetaminophen toxicity by acting as a glutathione precursor [14]. NAC was used in this patient because it also has a role in the treatment of non-acetaminophen-induced ALF (NAI-ALF) as its use has been shown to reduce mortality. A randomized control study of 80 patients with NAI-ALF (40 NAC; 40 placeboes) aimed at determining the mortality benefit of NAC in NAI-ALF showed that mortality was 53% in the control group and 28% in the NAC group. It was concluded that NAC use in NAI-ALF not only resulted in a mortality benefit but also decreased the length of hospital stay [15]. The literature regarding the benefit of NAC in remdesivir-associated ALF is limited but promising. For instance, in a case series by Carothers et al., two cases of remdesivir-associated ALF were presented and in both cases, NAC resulted in an improvement in ALF without any adverse effects [16]. The mechanism by which NAC improves outcomes in NAI-ALF is thought to be due to its antioxidant and vasodilatory properties resulting in increased renal circulation [17]. 

Other potential sources of DILI in this patient included lamotrigine and duloxetine, which were chronic medications for bipolar disorder and depression, respectively. Lamotrigine has rarely been linked to liver injury and liver failure; however, these cases are typically seen in infants and children and arise one to four weeks after starting the medication [18]. Although rare cases of duloxetine-induced liver injury have been documented in the literature, it is unlikely in our patient given the normal LFTs on admission before remdesivir was started. The Naranjo algorithm was utilized, which was developed to assess causality for adverse drug reactions, to further determine the probability of remdesivir being the cause of the DILI with subsequent ALF [19]. This case was deemed as a “probable” adverse drug reaction with a score of 5, further supporting our theory that this patient's ALF was likely due to an adverse effect of remdesivir in the absence of other causes.

## Conclusions

One of the leading causes of ALF in the United States is DILI. Many drugs have been linked to DILI; however, acetaminophen is the most common culprit. As described in this case, remdesivir can also, in rare cases, cause DILI. We conclude that this patient's ALF and subsequent death was caused by the use of remdesivir for the treatment of COVID-19. It is also important to note that NAC may have some therapeutic benefits in this subset of patients. During this novel era of COVID-19 infections and the rise in the use of remdesivir, it is important to note the potential adverse effects and outcomes this medication can have on patients with diverse medical histories. 
